# Opinion: An Existing Drug to Assess *In Vivo* for Potential Adjunctive Therapy of Ebola Virus Disease and Post-Ebola Syndrome

**DOI:** 10.3389/fphar.2019.01691

**Published:** 2020-01-30

**Authors:** Katherine Hendricks, Meredith Gilman Parrado, John Bradley

**Affiliations:** ^1^Independent researcher, Atlanta, GA, United States; ^2^Division of Infectious Diseases, Department of Pediatrics, UCSD School of Medicine, San Diego, CA, United States

**Keywords:** Ebola virus disease, minocycline, drug discovery, cytokine, chemokine, hepatoprotection

We currently have no approved drugs for the treatment of Ebola virus disease (EVD) or post-Ebola syndrome (PES). A substantial proportion of patients presenting for treatment die, including healthcare workers (HCWs) with hospital-acquired infections. More than 28,000 people were suspected or confirmed with EVD and 11,000 died in the West Africa outbreak during 2014–2016. A new EVD epicenter developed in the Democratic Republic of Congo in mid-2018, and it is likely that new outbreaks will occur in the future. The low survival rate discourages patients from presenting for treatment, and the occupational risk discourages HCWs from caring for highly infectious patients. A substantial proportion of survivors complain of chronic symptoms, such as eye problems (47%) and arthralgias (64%)—a condition that has been termed PES (Wilson et al., 2018). Inflammation may play a role in both the uveitis, which can result in blindness (Shantha et al., 2017) and in arthritis (Amissah-Arthur et al., 2017). Availability of an efficacious adjunctive treatment drug would save lives, increase the number of people presenting for treatment, and increase the willingness of HCWs to care for patients. An efficacious drug for adjunctive treatment of PES could decrease morbidity suffered by survivors. To date, most research efforts have focused on vaccine for prevention and either antivirals or antibody preparations for treatment. However, given the extensive inflammatory component of EVD, adjunctive therapy to decrease inflammation—but not globally downregulate the host immune response in a manner that could be detrimental (e.g., steroids)—may hold promise for better outcomes for those infected. Although drugs such as acetylsalicylic acid, ibuprofen, indomethacin, and celecoxib are also broadly anti-inflammatory, they all inhibit cyclooxygenase and can interfere with platelet aggregation—a characteristic that would be disqualifying for use with hemorrhagic fevers.

“Cytokine storm,” a burst in production of inflammatory cytokines, is thought by many to be integral to EVD pathogenesis, and high levels of tumor necrosis factor (TNF)-α, interleukin (IL)-6, IL-10, IL-1β, macrophage inflammatory protein (MIP)-1α, MIP-1β, and macrophage chemoattractant protein (MCP)-1 are associated with fatal infections (Ruibal et al., 2016; Vernet et al., 2017). Nuclear factor of activated T cells (NFAT) is thought to be *the* key transcriptional regulator of inflammatory mediators (Madelain et al., 2018). Knock-out mice with dampened cytokine response (Tim-1 -/-) are considerably more likely to survive Ebola virus challenge than their wild-type counterparts, despite a limited impact on viremia (Younan et al., 2017).

We questioned whether the inflammation associated with cytokine storm could be countered pharmaceutically—and having some familiarity with one antimicrobial (minocycline) with significant anti-inflammatory properties as well as documented antiviral activity—we searched PubMed for articles describing cytokine activity during EVD and during minocycline use.

Minocycline is an FDA-approved semisynthetic tetracycline with an established safety profile that has been used for 40 years in the treatment of acne and rosacea (Cullen and Cohan, 1976; Hersle and Gisslen, 1976), and more recently, for multidrug resistant *Acinetobacter* (Lashinsky et al., 2017). It appears to have activity against certain viral pathogens: It inhibits H7N9 replication *in vitro* (Josset et al., 2014), attenuates stimulation of interferon-related gene and TRAIL^Ψ^ in human dendritic cells and PBMCs exposed to HIV or influenza virus (Drewes et al., 2014), reduces West Nile Virus titers in brain-derived cell types in a dose-dependent manner (Michaelis et al., 2007), reduces Japanese encephalitis-induced damage in neuronal cell cultures (Mishra et al., 2009), and, based on molecular dynamics, may possibly inhibit the binding of Congo Crimean hemorrhagic fever virus to host nucleoprotein during cell infection—a host protein that is believed to be pivotal to viral replication (Sharifi et al., 2017). In a randomized controlled trial of patients with dengue hemorrhagic fever, compared to patients who received standard-of-care supportive treatment, those who also received the related tetracycline class antibiotic—doxycycline—had significantly lower mortality [20.9% vs 11.2% (p < 0.05)] and lower TNF and IL6 levels on days 3, 5, and 7 (p < 0.05 for all) (Fredeking et al., 2015). **Table 1** compares the effects of Ebola virus and minocycline on selected biomarkers including important cytokines and chemokines.

**Table 1:** Comparison of the Effect of Ebola Virus and Minocycline on Selected Biomarkers.

Biological Marker	Ebola Virus			Minocycline Effect on Physical or Infectious Challenge	
	Nonsurvivors	Survivors	Animal Model^§^	*In Vivo*	*In Vitro*
TNF-α	↑ (Baize et al., 2002)	↑ (Baize et al., 2002)	↑ (Geisbert et al., 2003; Mahanty et al., 2003; Rubins et al., 2007)	↓ (Ledeboer et al., 2005; Masocha et al., 2006; Suzuki et al., 2010; Wu et al., 2012; Hou et al., 2013; Ashraf et al., 2014)	↓ (Szeto et al., 2010; Tai et al., 2013)
IL-6	↑ (Baize et al., 2002; Hutchinson and Rollin, 2007; Wauquier et al., 2010)	↑ (Baize et al., 2002; Wauquier et al., 2010)	↑ (Geisbert et al., 2003; Rubins et al., 2007)	↓ (Ledeboer et al., 2005; Masocha et al., 2006; Suzuki et al., 2010)	↓ (Tai et al., 2013)
IFN-α	↑ (Villinger et al., 1999; Gupta et al., 2012)	↑ (Villinger et al., 1999; Gupta et al., 2012)	↑ (Geisbert et al., 2003; Mahanty et al., 2003)		↓ (Drewes et al., 2014)
MIP-1α	↑ (Wauquier et al., 2010)	↑ (Wauquier et al., 2010)	↑ (Geisbert et al., 2003; Rubins et al., 2007)	↓ (Suzuki et al., 2010)	↓ (Tai et al., 2013)
MIP-1β	↑ (Baize et al., 2002; Hutchinson and Rollin, 2007; Wauquier et al., 2010)	↑ (Wauquier et al., 2010)	↑ (Geisbert et al., 2003; Rubins et al., 2007)		↓ (Tai et al., 2013)
MCP-1			↑ (Geisbert et al., 2003; Mahanty et al., 2003; Rubins et al., 2007)	↓ (Suzuki et al., 2010)	↓ (Tai et al., 2013)
MMP-3			↑ (Cilloniz et al., 2011)	↓ (Masocha et al., 2006)	↓ (Fortier et al., 2010)
Markers of oxidative stress^¶^	↑ (Sanchez et al., 2004)		↑ (Hensley et al., 2002; Geisbert et al., 2003)	↓ (Suzuki et al., 2010; Huang et al., 2012; Ashraf et al., 2014)	
Pro-apoptotic factors/markers^Ψ^	↑ (Hutchinson and Rollin, 2007; Wauquier et al., 2010)	↑ (Hutchinson and Rollin, 2007)	↑ (Rubins et al., 2007)	↓ (Chu et al., 2005; Czerny et al., 2012; Drewes et al., 2014)	↓ (Yang et al., 2007; Tai et al., 2013)
Anti-apoptotic marker (bcl-2)			↓ (Gupta et al., 2012) ^(^*^in^^vitro^*^)^		↑ (Tang et al., 2007)
Liver function	↑ (Rollin et al., 2007)	↑ (Rollin et al., 2007)	↑ (Geisbert et al., 2003)	↓ (Chu et al., 2005; Czerny et al., 2012)	↓ (Szeto et al., 2010; Schwartz et al., 2013)

^§^In vitro data for Ebola can be found in references.

^¶^iNOS (inducible nitric oxide synthase), NO (nitric oxide), nitrate, SOD (superoxide dismutase).

^Ψ^TACE (tumor necrosis factor-α converting enzyme), TRAIL (tumor necrosis factor [TNF]-related apoptosis-inducing ligand), RANTES (regulated upon activation, normal T cell expressed and secreted), Eotaxin, Fas (Fas antigen), FasL (Fas antigen ligand), caspase-3, annexin.

IL, interleukin; MIP, macrophage inflammatory protein; MCP, macrophage chemoattractant protein; MMP, matrix metalloproteinase. ↑, significantly increased; ↓, significantly decreased.

As shown in our table, the anti-inflammatory activity of minocycline opposes those of many gene products of Ebola virus. It also selectively impairs NFAT-mediated transcriptional activation (Szeto et al., 2011). Due to its small size and lipophilic nature, minocycline may reach potentially therapeutic concentrations in tissue compartments for which antibiotic penetration is typically difficult, such as the eye (Abcouwer et al., 2013; Scholz et al., 2015) and joints (McEvoy, 2016). Such spaces appear to be capable of harboring Ebola virus (Varkey et al., 2015; Steptoe et al., 2017; Subissi et al., 2018) and are thought to contribute to the chronic sequelae seen in PES (Shantha et al., 2017; PREVAIL III Study Group, 2019; Heydari-Kamjani et al., 2019). However, pharmacokinetic/pharmacodynamic (PK/PD) data are lacking that would confirm minocycline penetration into such spaces. As previously mentioned, inflammation may play a role in both the potentially blinding uveitis and arthritis of PES. Although there are animal data to suggest that minocycline may have anti-inflammatory effects in the eye (Scholz et al., 2015) and human data to suggest anti-inflammatory activity in joints (Pradier et al., 2018), it is not known whether it has direct antiviral activity against Ebola virus.

Given minocycline’s broad anti-inflammatory activity against cytokines/chemokines that appear to be pathologically upregulated by Ebola virus and the safety history, relative availability, and affordability of minocycline, we feel it should be investigated as an adjunctive therapy in animal models of acute EVD. Given that it appears to cross into protected spaces where it may have anti-inflammatory activity, we feel it should also be investigated as adjunctive therapy for chronic sequelae of EVD (i.e., PES). It is likely that the anti-inflammatory benefit would be greater in certain infected populations (starting treatment early vs late in the infection), and it is also possible that, for some, downregulation of inflammation in general could impair host clearance of the virus. These important issues are amenable to investigation in animal models.

## Author Contributions

KH performed the literature review and drafted the table and text. MP helped to synthesize the tabular data and edited the text. JB reviewed the information and edited the text.

## Disclaimers

Use of trade names and commercial sources is for identification only and does not imply endorsement. This is an opinion piece based on literature review. No experiments have been conducted or data collected at this time for the potential adjunct treatment of Ebola virus disease or post-Ebola syndrome.

## Conflict of Interest

The authors declare that the research was conducted in the absence of any commercial or financial relationships that could be construed as a potential conflict of interest.

## References

[B1] Abcouwer S. F. Lin C. M. Shanmugam S. Muthusamy A. Barber A. J. Antonetti D. A. (2013). Minocycline prevents retinal inflammation and vascular permeability following ischemia-reperfusion injury. J. Neuroinflammation 10, 149. 10.1186/1742-2094-10-149 24325836 PMC3866619

[B2] Amissah-Arthur M. B. Poller B. Tunbridge A. Adebajo A. (2017). Musculoskeletal manifestations of Ebola virus. Rheumatology 57 (1), 28–31. 10.1093/rheumatology/kex082%JRheumatology 28379487

[B3] Ashraf T. Jiang W. Hoque M. T. Henderson J. Wu C. Bendayan R. (2014). Role of anti-inflammatory compounds in human immunodeficiency virus-1 glycoprotein120-mediated brain inflammation. J. Neuroinflammation 11, 91. 10.1186/1742-2094-11-91 24884548 PMC4046047

[B4] Baize S. Leroy E. M. Georges A. J. Georges-Courbot M. C. Capron M. Bedjabaga I. (2002). Inflammatory responses in Ebola virus-infected patients. Clin. Exp. Immunol. 128 (1), 163–168. 10.1046/j.1365-2249.2002.01800.x 11982604 PMC1906357

[B5] Chu H. C. Lin Y. L. Sytwu H. K. Lin S. H. Liao C. L. Chao Y. C. (2005). Effects of minocycline on Fas-mediated fulminant hepatitis in mice. Br. J. Pharmacol. 144 (2), 275–282. 10.1038/sj.bjp.0706079 15665864 PMC1576000

[B6] Cilloniz C. Ebihara H. Ni C. Neumann G. Korth M. J. Kelly S. M. (2011). Functional genomics reveals the induction of inflammatory response and metalloproteinase gene expression during lethal Ebola virus infection. J. Virol. 85 (17), 9060–9068. 10.1128/jvi.00659-11 21734050 PMC3165855

[B7] Cullen S. I. Cohan R. H. (1976). Minocycline therapy in acne vulgaris. Cutis 17 (6), 1208–1210. 14. 138560

[B8] Czerny C. Kholmukhamedov A. Theruvath T. P. Maldonado E. N. Ramshesh V. K. Lehnert M. (2012). Minocycline decreases liver injury after hemorrhagic shock and resuscitation in mice. HPB Surg. World J. Hepatic Pancreatic Biliary Surg. 2012, 259512. 10.1155/2012/259512 PMC3375163 22719175

[B9] Drewes J. L. Szeto G. L. Engle E. L. Liao Z. Shearer G. M. Zink M. C. (2014). Attenuation of pathogenic immune responses during infection with human and simian immunodeficiency virus (HIV/SIV) by the tetracycline derivative minocycline. PloS One 9 (4), e94375. 10.1371/journal.pone.0094375 24732038 PMC3986096

[B10] Fortier L. A. Motta T. Greenwald R. A. Divers T. J. Mayr K. G. (2010). Synoviocytes are more sensitive than cartilage to the effects of minocycline and doxycycline on IL-1alpha and MMP-13-induced catabolic gene responses. J. Orthopaedic Res. Off. Publ. Orthopaedic Res. Soc. 28 (4), 522–528. 10.1002/jor.21006 19813262

[B11] Fredeking T. M. Zavala-Castro J. E. Gonzalez-Martinez P. Moguel-Rodriguez W. Sanchez E. C. Foster M. J. (2015). Dengue patients treated with doxycycline showed lower mortality associated to a reduction in IL-6 and TNF levels. Recent patents on anti-infective drug discovery. Recent Pat. Antiinfect. Drug Discov. 10, 1, 51–58. 10.2174/1574891x10666150410153839 25858261

[B12] Geisbert T. W. Hensley L. E. Larsen T. Young H. A. Reed D. S. Geisbert J. B. (2003). Pathogenesis of Ebola hemorrhagic fever in cynomolgus macaques: evidence that dendritic cells are early and sustained targets of infection. Am. J. Pathol. 163 (6), 2347–2370. 10.1016/s0002-9440(10)63591-2 14633608 PMC1892369

[B13] Gupta M. MacNeil A. Reed Z. D. Rollin P. E. Spiropoulou C. F. (2012). Serology and cytokine profiles in patients infected with the newly discovered Bundibugyo ebolavirus. Virology 423 (2), 119–124. 10.1016/j.virol.2011.11.027 22197674

[B14] Hensley L. E. Young H. A. Jahrling P. B. Geisbert T. W. (2002). Proinflammatory response during Ebola virus infection of primate models: possible involvement of the tumor necrosis factor receptor superfamily. Immunol. Lett. 80 (3), 169–179. 10.1016/s0165-2478(01)00327-3 11803049

[B15] Hersle K. Gisslen H. (1976). Minocycline in acne vulgaris: a double-blind study. Curr. Ther. Res. Clin. Exp. 19 (3), 339–342. 131678

[B16] Heydari-Kamjani M. Demory Beckler M. Kesselman M. M. (2019). Reconsidering the use of minocycline in the preliminary treatment regime of rheumatoid arthritis. Cureus 11 (8), e5351. 10.7759/cureus.5351 31608186 PMC6783212

[B17] Hou Y. Ryu C. H. Park K. Y. Kim S. M. Jeong C. H. Jeun S. S. (2013). Effective combination of human bone marrow mesenchymal stem cells and minocycline in experimental autoimmune encephalomyelitis mice. Stem Cell Res. Ther. 4 (4), 77. 10.1186/scrt228 23826999 PMC3854709

[B18] Huang C. Li R. Zeng Q. Ding Y. Zou Y. Mao X. (2012). Effect of minocycline postconditioning and ischemic postconditioning on myocardial ischemia-reperfusion injury in atherosclerosis rabbits. J. Huazhong University Sci. Technol. Med. Sci. = Hua zhong ke ji da xue xue bao Yi xue Ying De wen ban = Huazhong keji daxue xuebao Yixue Yingdewen ban 32 (4), 524–529. 10.1007/s11596-012-0090-y 22886964

[B19] Hutchinson K. L. Rollin P. E. (2007). Cytokine and chemokine expression in humans infected with Sudan Ebola virus. J. Infect. Dis. 196 Suppl 2, S357–S363. 10.1086/520611 17940971

[B20] Josset L. Zeng H. Kelly S. M. Tumpey T. M. Katze M. G. (2014). Transcriptomic characterization of the novel avian-origin influenza A (H7N9) virus: specific host response and responses intermediate between avian (H5N1 and H7N7) and human (H3N2) viruses and implications for treatment options. mBio 5 (1), e01102–e01113. 10.1128/mBio.01102-13 24496798 PMC3950506

[B21] Lashinsky J. N. Henig O. Pogue J. M. Kaye K. S. (2017). Minocycline for the treatment of multidrug and extensively drug-resistant a. baumannii: A review. Infect. Dis. Ther. 6 (2), 199–211. 10.1007/s40121-017-0153-2 28357705 PMC5446366

[B22] Ledeboer A. Sloane E. M. Milligan E. D. Frank M. G. Mahony J. H. Maier S. F. (2005). Minocycline attenuates mechanical allodynia and proinflammatory cytokine expression in rat models of pain facilitation. Pain 115 (1-2), 71–83. 10.1016/j.pain.2005.02.009 15836971

[B23] Madelain V. Baize S. Jacquot F. Reynard S. Fizet A. Barron S. (2018). Ebola viral dynamics in nonhuman primates provides insights into virus immuno-pathogenesis and antiviral strategies. Nat. Commun. 9 (1), 4013. 10.1038/s41467-018-06215-z 30275474 PMC6167368

[B24] Mahanty S. Gupta M. Paragas J. Bray M. Ahmed R. Rollin P. E. (2003). Protection from lethal infection is determined by innate immune responses in a mouse model of Ebola virus infection. Virology 312 (2), 415–424. 10.1016/s0042-6822(03)00233-2 12919746

[B25] Masocha W. Rottenberg M. E. Kristensson K. (2006). Minocycline impedes African trypanosome invasion of the brain in a murine model. Antimicrob. Agents Chemother. 50 (5), 1798–1804. 10.1128/aac.50.5.1798-1804.2006 16641452 PMC1472198

[B26] McEvoy T. (2016). Minocycline: rheumatoid arthritis. Hospital Pharm. 51 (7), 535–538. 10.1310/hpj5107-535 PMC4981101 27559186

[B27] Michaelis M. Kleinschmidt M. C. Doerr H. W. Cinatl J. Jr. (2007). Minocycline inhibits West Nile virus replication and apoptosis in human neuronal cells. J. Antimicrob. Chemother. 60 (5), 981–986. 10.1093/jac/dkm307 17872917

[B28] Mishra M. K. Ghosh D. Duseja R. Basu A. (2009). Antioxidant potential of minocycline in japanese encephalitis virus infection in murine neuroblastoma cells: correlation with membrane fluidity and cell death. Neurochem. Int. 54 (7), 464–470. 10.1016/j.neuint.2009.01.022 19428790

[B29] Pradier M. Robineau O. Boucher A. Titecat M. Blondiaux N. Valette M. (2018). Suppressive antibiotic therapy with oral tetracyclines for prosthetic joint infections: a retrospective study of 78 patients. Rheumatology 46 (1), 39–47. 10.1007/s15010-017-1077-1 29052797

[B30] PREVAIL III Study Group (2019). A longitudinal study of ebola sequelae in liberia. N. Engl. J. Med. 380, 10, 924–934. 10.1056/NEJMoa1805435 30855742 PMC6478393

[B31] Rollin P. E. Bausch D. G. Sanchez A. (2007). Blood chemistry measurements and D-Dimer levels associated with fatal and nonfatal outcomes in humans infected with Sudan Ebola virus. J. Infect. Dis. 196 Suppl 2, S364–S371. 10.1086/520613 17940972

[B32] Rubins K. H. Hensley L. E. Wahl-Jensen V. Daddario DiCaprio K. M. Young H. A. Reed D. S. (2007). The temporal program of peripheral blood gene expression in the response of nonhuman primates to Ebola hemorrhagic fever. Genome Biol. 8 (8), R174. 10.1186/gb-2007-8-8-r174 17725815 PMC2375004

[B33] Ruibal P. Oestereich L. Ludtke A. Becker-Ziaja B. Wozniak D. M. Kerber R. (2016). Unique human immune signature of Ebola virus disease in Guinea. Nature 533 (7601), 100–104. 10.1038/nature17949 27147028 PMC4876960

[B34] Sanchez A. Lukwiya M. Bausch D. Mahanty S. Sanchez A. J. Wagoner K. D. (2004). Analysis of human peripheral blood samples from fatal and nonfatal cases of Ebola (Sudan) hemorrhagic fever: cellular responses, virus load, and nitric oxide levels. J. Virol. 78 (19), 10370–10377. 10.1128/jvi.78.19.10370-10377.2004 15367603 PMC516433

[B35] Scholz R. Sobotka M. Caramoy A. Stempfl T. Moehle C. Langmann T. (2015). Minocycline counter-regulates pro-inflammatory microglia responses in the retina and protects from degeneration. J. Neuroinflammation 12, 209. 10.1186/s12974-015-0431-4 26576678 PMC4650866

[B36] Schwartz J. Holmuhamedov E. Zhang X. Lovelace G. L. Smith C. D. Lemasters J. J. (2013). Minocycline and doxycycline, but not other tetracycline-derived compounds, protect liver cells from chemical hypoxia and ischemia/reperfusion injury by inhibition of the mitochondrial calcium uniporter. Toxicol. Appl. Pharmacol. 273 (1), 172–179. 10.1016/j.taap.2013.08.027 24012766 PMC4402227

[B37] Shantha J. G. Crozier I. Yeh S. (2017). An update on ocular complications of Ebola virus disease. Curr. Opin. Ophthalmol. 28 (6), 600–606. 10.1097/icu.0000000000000426 28872492 PMC5988239

[B38] Sharifi A. Amanlou A. Moosavi-Movahedi F. Golestanian S. Amanlou M. (2017). Tetracyclines as a potential antiviral therapy against Crimean Congo hemorrhagic fever virus: Docking and molecular dynamic studies. Comput. Biol. Chem. 70, 1–6. 10.1016/j.compbiolchem.2017.06.003 28709136

[B39] Steptoe P. J. Scott J. T. Baxter J. M. Parkes C. K. Dwivedi R. Czanner G. (2017). Novel retinal lesion in ebola survivors, sierra leone, 2016. Emerging Infect. Dis. 23 (7), 1102–1109. 10.3201/eid2307.161608 28628441 PMC5512503

[B40] Subissi L. Keita M. Mesfin S. Rezza G. Diallo B. Van Gucht S. (2018). Ebola virus transmission caused by persistently infected survivors of the 2014-2016 outbreak in west africa. J. Infect. Dis. 218 (suppl_5), S287–Ss91. 10.1093/infdis/jiy280 29920602 PMC6249578

[B41] Suzuki H. Sugimura Y. Iwama S. Suzuki H. Nobuaki O. Nagasaki H. (2010). Minocycline prevents osmotic demyelination syndrome by inhibiting the activation of microglia. J. Am. Soc. Nephrol. JASN 21 (12), 2090–2098. 10.1681/asn.2010040438 21030598 PMC3014022

[B42] Szeto G. L. Brice A. K. Yang H. C. Barber S. A. Siliciano R. F. Clements J. E. (2010). Minocycline attenuates HIV infection and reactivation by suppressing cellular activation in human CD4+ T cells. J. Infect. Dis. 201 (8), 1132–1140. 10.1086/651277 20205570 PMC3739045

[B43] Szeto G. L. Pomerantz J. L. Graham D. R. Clements J. E. (2011). Minocycline suppresses activation of nuclear factor of activated T cells 1 (NFAT1) in human CD4+ T cells. J. Biol. Chem. 286 (13), 11275–11282. 10.1074/jbc.M110.210518 21282105 PMC3064183

[B44] Tai K. Iwasaki H. Ikegaya S. Ueda T. (2013). Minocycline modulates cytokine and chemokine production in lipopolysaccharide-stimulated THP-1 monocytic cells by inhibiting IkappaB kinase alpha/beta phosphorylation. Transl. Res. J. Lab. Clin. Med. 161 (2), 99–109. 10.1016/j.trsl.2012.10.001 23108365

[B45] Tang X. N. Wang Q. Koike M. A. Cheng D. Goris M. L. Blankenberg F. G. (2007). Monitoring the protective effects of minocycline treatment with radiolabeled annexin V in an experimental model of focal cerebral ischemia. J. Nucl. Med. Off. Publ. Soc. Nucl. Med. 48 (11), 1822–1828. 10.2967/jnumed.107.041335 17942809

[B46] Varkey J. B. Shantha J. G. Crozier I. Kraft C. S. Lyon G. M. Mehta A. K. (2015). Persistence of ebola virus in ocular fluid during convalescence. N. Engl. J. Med. 372 (25), 2423–2427. 10.1056/NEJMoa1500306 25950269 PMC4547451

[B47] Vernet M. A. Reynard S. Fizet A. Schaeffer J. Pannetier D. Guedj J. (2017). Clinical, virological, and biological parameters associated with outcomes of Ebola virus infection in Macenta, Guinea. JCI Insight 2 (6), e88864. 10.1172/jci.insight.88864 28352651 PMC5358491

[B48] Villinger F. Rollin P. E. Brar S. S. Chikkala N. F. Winter J. Sundstrom J. B. (1999). Markedly elevated levels of interferon (IFN)-gamma, IFN-alpha, interleukin (IL)-2, IL-10, and tumor necrosis factor-alpha associated with fatal Ebola virus infection. J. Infect. Dis. 179 Suppl;1, S188–S191. 10.1086/514283 9988183

[B49] Wauquier N. Becquart P. Padilla C. Baize S. Leroy E. M. (2010). Human fatal zaire ebola virus infection is associated with an aberrant innate immunity and with massive lymphocyte apoptosis. PLoS Negl. Trop. Dis. 4 (10), e837. 10.1371/journal.pntd.0000837 20957152 PMC2950153

[B50] Wilson H. W. Amo-Addae M. Kenu E. Ilesanmi O. S. Ameme D. K. Sackey S. O. (2018). Post-ebola syndrome among ebola virus disease survivors in montserrado county, liberia 2016. BioMed Res. Int. 2018, 1909410. 10.1155/2018/1909410 30050920 PMC6046154

[B51] Wu K. L. Chan S. H. Chan J. Y. (2012). Neuroinflammation and oxidative stress in rostral ventrolateral medulla contribute to neurogenic hypertension induced by systemic inflammation. J. Neuroinflammation 9, 212. 10.1186/1742-2094-9-212 22958438 PMC3462714

[B52] Yang L. P. Zhu X. A. Tso M. O. (2007). Minocycline and sulforaphane inhibited lipopolysaccharide-mediated retinal microglial activation. Mol. Vision 13, 1083–1093. PMC2779142 17653053

[B53] Younan P. Iampietro M. Nishida A. Ramanathan P. Santos R. I. Dutta M. (2017). Ebola virus binding to Tim-1 on T lymphocytes induces a cytokine storm. mBio 8 (5), e00845–17. 10.1128/mBio.00845-17 28951472 PMC5615193

